# Epidemiology and Risk Factors of COVID-19-Related Mortality

**DOI:** 10.7759/cureus.20072

**Published:** 2021-12-01

**Authors:** Debarchan Barman Roy, Vandana Gupta, Shalini Tomar, Gaurav Gupta, Ashutosh Biswas, Piyush Ranjan, Upendra Baitha, Shivam Pandey, Bindoo Prakash, Naveet Wig

**Affiliations:** 1 Medicine, All India Institute of Medical Sciences, New Delhi, New Delhi, IND; 2 Periodontics, All India Institute of Medical Sciences, New Delhi, New Delhi, IND; 3 Biostatistics, All India Institute of Medical Sciences, New Delhi, New Delhi, IND

**Keywords:** covid-19 mortality, diabetes and hypertension, risk-factors, epidemiology and public health, covid-19 india

## Abstract

Introduction

During the coronavirus disease 2019 (COVID-19) pandemic in India, several characteristics of hospitalized COVID-19 patients, based on demographics, mortality predictors, and presence of comorbidities, were found to be associated with poor outcomes. The objective of this study was to identify such epidemiological and clinical characteristics among the patients admitted at a tertiary-care center in India that may have predisposed them to COVID-19-related mortality.

Methods

This retrospective observational study conducted at the Department of Medicine, All India Institute of Medical Sciences, New Delhi, in May 2021 included 141 COVID-19 confirmed patients. The medical history, demographic characteristics, comorbidities, clinical findings, and laboratory data of each patient were obtained. The data were analyzed to identify significant clinical and laboratory parameters that led to the adverse final outcomes.

Results

Hypertension was the most common comorbidity and the presence of diabetes with hypertension led to poorer final outcomes. Lower oxygen saturation and requirement of oxygen supplementation at admission along with worse prognostic scores during admission led to poorer outcomes. Twenty-seven patients needed non-invasive ventilation (NIV) during the hospital course, and all ultimately landed up among the 56 patients who were managed on invasive mechanical ventilation (IMV). Multivariate logistic regression analysis performed identified COVID-19 severity at admission, co-existence of hypertension and diabetes mellitus, systolic blood pressure less than 90 mm Hg, and serum creatinine greater than 1.2 mg/dL to be associated with higher COVID-19 mortality.

Conclusion

COVID-19 patients having the co-existence of diabetes and hypertension constitute a high-risk group and may be targeted by prompt vaccination strategies. The presence of severe disease along with a need for oxygen therapy and other intensive care interventions ultimately led to unfavorable outcomes.

## Introduction

India witnessed the second wave of coronavirus disease 2019 (COVID-19) in March 2021, with 30 million confirmed cases and almost 0.4 million mortalities [1]. Several epidemiological studies from China, Europe, North America, Brazil, and India identified various characteristics of hospitalized COVID-19 patients based on demographics, mortality predictors, and association with comorbidities, which were associated with poor outcomes [2-4]. The majority of the studies have focussed on respiratory outcomes, mortality, risk factors, steroid use while a few have reported the multisystem outcomes and predictors of mortality mostly for critically ill COVID-19 patients. Mortality was extremely high in patients who were suffering from a severe form of the disease and with multiple comorbid [5-6]. Similarly, the use of various mortality predictors to prognosticate and approximate the further clinical course of illness in a COVID-19 patient is not well-established. In this small retrospective study, we compared various baseline demographic and clinical characteristics along with laboratory data and clinical outcomes in both survivor and non-survivor COVID-19-confirmed patients admitted to our center.

## Materials and methods

Study design

This was a retrospective observational study of COVID-19 patients admitted to the Department of Medicine, All India Institute of Medical Sciences, New Delhi, in May 2021. The study included all patients who were 18 years or older, with severe acute respiratory syndrome coronavirus 2 (SARS-CoV-2) infection confirmed by a positive reverse-transcriptase-polymerase chain reaction (RT-PCR) assay or a cartridge-based nucleic acid amplification test (CB-NAAT) of the nasopharyngeal swab samples collected during admission. The study protocol was approved by the Ethics Committee of the All India Institute of Medical Sciences, New Delhi, with IEC number: IEC- 295/17.04.2020, RP-37/2020.

Clinical and biological data

In this study, the medical history, demographic characteristics, comorbidities, clinical findings, and laboratory data of each patient were recorded. The COVID-19 severity class along with Acute Physiology and Chronic Health Evaluation (APACHE) II, Sequential Organ Failure Assessment (SOFA), and Modified Early Warning Score (MEWS) scores at admission were considered. The baseline parameters and further hospital course were reviewed. Patient survival was the primary outcome. The final outcome of these patients was categorized as either survivor or non-survivor.

Statistical analysis

For categorical data, results were summarized as the actual number of patients (n) and percentages (cumulative incidence). All continuous variables were expressed as mean with standard deviation or median with inter-quartile range. Individual parameters among survivors and non-survivors were compared using the chi-square test or Fisher’s exact test for categorical variables, independent t-test for continuous variables following normal distribution, and Wilcoxon rank-sum test for parameters following non-normal distribution. Univariable and then multivariable backward stepwise logistic regression analyses were performed to identify independent predictors of hospital mortality. A p-value of <0.05 was considered significant. All statistical analysis was conducted using StataCorp. 2019 (Stata Statistical Software: Release 16. College Station, TX: StataCorp LLC).

## Results

Demographic and clinical features

A total of 141 patients were included in the study. Their baseline demographic characteristics and clinical features are shown in Table 1. During admission, 81 (57.4%) patients had some comorbidity like hypertension (58, 41.1%), diabetes (53, 37.6%), or hypothyroidism (18, 12.8%) while 33 (23.4%) had diabetes with hypertension.

**Table 1:** Baseline demographic and clinical characteristics of the patients APACHE=Acute Physiology and Chronic Health Evaluation; SOFA=Sequential Organ Failure Assessment; MEWS=Modified Early Warning Score

Patient characteristics {n (%) or mean ± SD or Median (IQR)}	Total patients (n=141)	Survivors (n=87)	Non-survivors(n=54)	p-value
Age (years)				
Age<50yrs	66(46.8%)	49(56.3%)	17(31.5%)	0.004
Age>50yrs	75(53.2%)	38(43.7%)	37(68.5%)	0.004
Gender				
Male	80(56.7%)	48(55.2%)	32(59.3%)	0.634
Female	61(43.3%)	39(44.83%)	22(40.7%)	0.634
Any comorbidities	81(57.4%)	44(50.6%)	37(68.5%)	0.036
Diabetes mellitus	53 (37.6%)	29(33.3%)	24(44.4%)	0.185
Hypertension	58(41.1%)	27(31%)	31(57.4%)	0.002
Diabetes + Hypertension	33(23.4%)	14(16.1%)	19(35.2%)	0.009
Coronary artery disease	7(4.9%)	5(5.8%)	2(3.7%)	0.587
Chronic kidney disease	8(5.7%)	4(4.6%)	4(7.4%)	0.483
Chronic obstructive pulmonary disease	2(1.4%)	-	2(3.7%)	0.072
Hypothyroidism	18(12.8%)	10(11.5%)	8(14.8%)	0.566
Initial vital signs				
Temperature >38˚C	17(12.1%)	11(12.8%)	6(11.1%)	0.767
% Oxygen Saturation	93±9	95±4	88±12	0.682
Oxygen saturation <90%	91(65%)	40(46.5%)	51(94.4%)	0.001
Need for oxygen supplementation at admission triage	89(63.1%)	40(45.9%)	49(90.7%)	0.001
Respiratory rate >22/min	87(61.7%)	42(48.3%)	45(83.3%)	0.001
Heart rate > 100/min	67(47.5%)	23(29.9%)	41(75.9%)	0.001
Systolic blood pressure in mmHg	123±24	125±19	123±25	0.065
Systolic blood pressure (SBP) ≤90 mmHg	15(10.6%)	6(6.9%)	9(16.7%)	0.067
Diastolic blood pressure in mmHg	75 ± 16	75±12	74±20	0.065
COVID-19 severity class at admission				
Mild	38(26.9%)	36(41.4%)	2(3.7%)	0.001
Moderate	25(17.7%)	19(21.8%)	6(11.1%)	0.001
Severe	78(55.3%)	32(36.8%)	46(85.2%)	0.001
APACHE II score	9(0-36)	5 (3-9)	11 (9-17)	0.001
APACHE II score ≥10	53(37.6%)	19(21.8%)	34(62.9%)	0.001
SOFA score	1 (1-3)	1 (0-1)	1 (1-3)	0.001
MEWS score	2.9±1.9	2±1. 1	3.9±1.9	0.001
MEWS score ≥ 5	26(18.4%)	9(10.3%)	17(31.5%)	0.002

Non-survivors had a lower mean oxygen saturation on pulse oximetry (SpO2) at admission compared to survivors (88%±12% vs 95%±4%; p-value=0.682). Out of 54 non-survivors, 51 (94.4%) were admitted with a SpO2<90%, which was statistically significant {51(94.4%) vs 40(46.5%); p-value<0.001}. At admission, 89 (63.1%) patients required oxygen supplementation and 49 (90.7%) of them succumbed to their illness. The COVID-19 severity class and prognostic scores of APACHE II, SOFA, and MEWS were all significantly worse in the non-survivor group (p-value <0.001).

Laboratory investigations

As listed in Table 2, non-survivors had a significantly higher mean total leukocyte count and median serum alkaline phosphatase, serum urea, serum creatinine, and serum lactate dehydrogenase. The significantly elevated markers of inflammation among non-survivors were C-reactive protein and serum ferritin.

**Table 2:** Baseline laboratory parameters and interventions in the patients ICU=intensive care unit; NIV=non-invasive ventilation; IMV=invasive mechanical ventilation; P:F ratio=SpO2:FiO2 ratio

Patient characteristics {n (%) or Mean +/- SD or Median (IQR)}	Total patients (n=141)	Survivors (n=87)	Non-survivors (n=54)	p-value
Hemoglobin (g/dL)	10.98±3.8	11.63±3.64	10.57±3.82	0.93
Total Leukocyte Count (cells/µL)	11229±5679	9973±4912	13252±6268	0.001
Platelets (cells/µL)	202237 (108180-278200)	175000 (130000-250000)	204500 (148000-254000)	0.448
Total Bilirubin (mg/dL)	0.91 (0.68-1.8)	0.56 (0.37-0.82)	0.6 (0.38-1.1)	0.298
Aspartate Aminotransferase (U/L)	61.88 (25.3-78.3)	27.5 (14.5-52.5)	34 (22-49)	0.318
Alanine Aminotransferase (U/L)	89.31 (19-111.2)	65.5 (17-112.5)	53 (24-76)	0.295
Serum Alkaline Phosphatase (U/L)	75.72 (26-98.3)	36 (17-68.5)	83 (47-143)	0.001
Serum Urea (mg/dL)	50 (24-62)	26 (22-47)	52.5 (35-93)	0.001
Serum Creatinine (mg/dL)	1.2 (0.6-1.1)	0.7 (0.5-0.9)	0.8 (0.6-1.6)	0.007
Serum Sodium (mmol/L)	137.65±13.7	135.9±15.9	140.2±8.8	0.185
Serum Potassium (mmol/L)	4.46±0.98	4.41±0.98	4.52±0.99	0.126
Serum Calcium (mg/dL)	8.59±3.67	8.1±2.64	8.05±0.68	0.551
Serum Phosphate (mg/dL)	3.54 ± 1.54	3.32±0.88	4.0±2.0	0.042
Serum Lactate Dehydrogenase (U/L)	524.65 (326.8-965.2)	352.5 (262-449)	640 (514-807.5)	0.001
Capillary Blood Glucose (mg/dL)	163 (96-184)	140 (101-226)	148 (111-210)	0.001
Glycated Hemoglobin (HbA1c) (%)	7.59 ± 2.1	7.77±2.29	7.51±1.62	0.356
Prothrombin Time (seconds)	14.09 ± 4.44	13.38±3.08	15.39±5.28	0.256
Activated Partial Thromboplastin Time (seconds)	32.41 (12.3-37.4)	29.97 (16.2-33.15)	31.4 (25.6-36)	0.241
Fibrinogen (mg/dL)	438.14 (112.3-645.3)	444.2 (298.1-596)	474.6 (119.1-718.2)	0.945
D-Dimer (ng/mL)	119.35 (15-415.5)	241.1 (15-415.5)	15 (15-315.6)	0.024
Procalcitonin (ng/mL)	0.63 (0.36-3.54)	0.47 (0.23-0.7)	0.85 (0.4-3.67)	0.045
Lactic Acid (mmol/L)	1.75 (1.82-2.1)	1.63 (1.21-1.9)	1.7 (1.3-2.1)	0.007
C-reactive Protein (CRP) (mg/L)	79.59 (33.7-101.5)	28.1 (12.45-72.15)	94 (44.2-196.2)	0.001
Serum Ferritin (ng/mL)	833.38 (143-1423.1)	401.5 (133-824)	823 (198-1649)	0.012
Interleukin-6 (pg/mL)	94.73 (16.8-119.2)	1.3 (0.9-1.8)	47.2 (29-128.6)	0.061
Intensive Care Management				
Renal Replacement Therapy	21 (15%)	1(1.2%)	20 (37%)	0.001
Vasopressor Use	25 (17.7%)	-	25 (46.3%)	0.001
Prone Positioning	14 (9.9%)	-	14 (25.9%)	0.001
Tracheostomy	1 (0.7%)	-	1 (1.8%)	0.203
Non-Invasive Ventilation (NIV)	27 (19.2%)	-	27 (50%)	0.001
Days of NIV (n=27)	1 (0-3)	-	1 (0-3)	0.001
Invasive Mechanical Ventilation (IMV)	56 (39.7%)	2 (2.3%)	54 (100%)	0.001
Days of IMV (n=56)	5 (2-7)	4 (1-6)	4 (2-8)	0.912
P:F Ratio (baseline)	80 (70-110)	-	80 (70-110)	0.092
Time From Hospital Admission to Invasive Ventilation in Days	2 (1-6)	-	2 (1-6)	0.001
Time From Symptom Onset to Invasive Ventilation in Days	5 (2-11)	0 (0-4)	7.5 (3-13)	0.001

Management and intervention

Twenty-seven patients (19.2%) needed non-invasive ventilation (NIV) and 56 patients (39.7%) were managed on invasive mechanical ventilation (IMV) during the hospital stay. All 27 of these NIV patients eventually needed IMV. All the 54 non-survivor patients were managed on IMV during their stay while only two patients (2.3%) among the survivors were managed on IMV (p-value < 0.001). Prone positioning was done in 14 patients (9.9%), 25 patients (17.7%) needed use of vasopressor, 21 patients (15%) needed renal replacement therapy while percutaneous tracheostomy was performed in only one patient (0.7%).

Multivariate logistic regression analysis of mortality in COVID-19 patients

A multivariate logistic regression analysis was performed by considering the variables of significance as shown in Table 3. The variables identified to be independently associated with a statistically significant higher risk of mortality in COVID-19 disease were COVID-19 severity at admission, co-existence of hypertension and diabetes mellitus, systolic blood pressure less than 90 mmHg, and serum creatinine greater than 1.2 mg/dL.

**Table 3:** Multivariate logistic regression analysis of mortality in COVID-19 patients SBP=systolic blood pressure; LDH=lactate dehydrogenase; CRP=C-reactive protein

Covariates	Number (%)	Unadjusted OR (95% CI)	p-value	Adjusted OR (95% CI)	p-value
Male	80(56.7%)	0.846(0.425-1.683)	0.634	-	-
COVID-19 severity at admission	141(100%)	3.684(0.827-16.416)	0.087	5.431(1.566-18.836)	0.008
Diabetes + Hypertension	33(23.4%)	2.714(1.222-6.028)	0.014	3.88(1.0-15.052)	0.050
Temperature >38˚C	17(12.1%)	0.852(0.296-2.457)	0.767	-	-
Oxygen saturation <90%	91(65%)	19.55(5.662-67.496)	0.001	-	-
Respiratory rate > 22/min	87(61.7%)	5.357(2.336-12.286)	0.001	-	-
Heart rate > 100/min	67(47.5%)	7.399(3.410-16.054)	0.001	-	-
Systolic blood pressure (SBP) ≤90 mmHg	15(10.6%)	3.6(1.158-11.185)	0.027	33.291(2.697-410.903)	0.006
COVID-19 severity score at admission	141(100%)	4.912(2.617-9.221)	0.001	-	-
Hemoglobin < 12.0 g/dL	72(51.1%)	1.709(0.859-3.398)	0.126	-	-
Total leukocyte count > 11000 cells/µL	72(51.1%)	2.833(1.395-5.755)	0.004	-	-
Creatinine > 1.2 mg/dL	27(19.3%)	5.293(2.115-13.245)	0.001	6.695(1.305-34.354)	0.023
Phosphate > 4.5 mg/dL	24(17.6%)	5.127(1.952-13.463)	0.001	-	-
LDH > 214 U/L	99(86.8%)	1.864(0.554-6.269)	0.314	-	-
D-dimer > 255 ng/mL	47(37.9%)	0.593(0.277-1.268)	0.178	-	-
CRP > 5 mg/L	119(92.2%)	6.084(0.746-49.597)	0.092	-	-
Ferritin > 150 ng/mL	90(72%)	2.111(0.888-5.018)	0.091	-	-
Duration of hospital stay (days)	141(100%)	0.94(0.475-1.860)	0.859	-	-

## Discussion

We conducted this small-scale, retrospective study and analyzed risk factors among these patients. Non-survivors had a higher mean age compared to survivors, similar to other studies from Wuhan and Pakistan [7-8]. More than half of the patients had some underlying chronic illness, commonly hypertension followed by diabetes and hypothyroidism, similar to studies on Chinese and Italian patients [7,9]. The meta-analysis by Sahni S et al., including more than 20000 patients from around the world, reported the same [10]. However, a South Indian study showed contrasting results, with diabetes more prevalent than hypertension [11]. One-fourth of our patients had both hypertension and diabetes significantly more than a Wuhan study where 6.7% of patients had both comorbidities [12].

The mean SpO2 recorded during admission was significantly lower among non-survivors, with 94% of them having SpO2 <90%, thus signifying that a significant majority of the non-survivors had severe COVID-19 disease at admission. Coincidentally, almost 90% of the non-survivors needed oxygen supplementation at the time of admission. This is in conjunction with similar studies where a majority of non-survivors had a lower SpO2 compared to the survivor group and had an early need of supplemental oxygen [11-14]. A study with ICU patients in Florida with a similar sample size showed 50% of non-survivors had SpO2 <90% during admission while 60% were in need of supplemental oxygen. In comparison, our non-survivors could be considered to have more severe COVID-19 disease. Similarly, the COVID-19 severity class along with the prognostic scores of APACHE II, SOFA, and MEWS score were all worse in the non-survivor group like various previous studies [13,15-16].

Considering the various laboratory parameters, the non-survivor group had elevated mean total leukocyte count, serum urea, serum creatinine, serum alkaline phosphatase, and serum lactate dehydrogenase compared to the survivor group. The various other parameters were, however, almost similar in both groups. This is in conjunction with most other studies, thus signifying the role of these laboratory parameters in determining the severity of COVID-19 disease and the high rates of mortality [8,13,17-18]. C-reactive protein and serum ferritin were elevated among non-survivors thus stressing the role of the inflammatory cytokine storm in the disease severity and poor prognosis [13].

Around 20% of our patients had severe respiratory distress and needed NIV management. Nevertheless, all these patients clinically deteriorated subsequently and were further managed on IMV. Thus, around 40% of our patients were managed on IMV and only two of them survived while the rest all succumbed to their illness. This was different compared to the study by Menzella F et al. where around 48% of patients were managed on NIV and the mortality rate recorded was 25% [19]. Around 10% of our patients were managed with prone positioning. This number was fewer than similar studies but our resource-poor setting had a role to play [20].

We performed a multivariate logistic regression analysis considering the variables that showed significance. A higher COVID severity at admission, co-existing hypertension and diabetes, lower systolic blood pressure at admission, and elevated serum creatinine were all identified to be significant independent risk factors for mortality in our regression model. Our findings could be considered in conjunction with various previous similar studies [12-13]. However, further studies with a greater sample size are suggested to better correlate these findings.

Our study has highlighted several important aspects but the interpretation should be done taking into account several limitations. The study was retrospective with a small sample size. It didn’t consider the effect of various therapies namely antibiotics, anti-inflammatory drugs, and other supportive therapies on the results. Additionally, while examining multiple factors associated with survival using multivariate regression analysis, there may have been unidentified potential confounders.

## Conclusions

Our study has highlighted several important aspects during the second COVID-19 wave in the Indian subcontinent. About 63% of our patients needed oxygen therapy at presentation, and 40% needed mechanical ventilation at some point of time during hospitalization. Both diabetes and hypertension were associated with a substantial increase in the risk of COVID-19 mortality. Our findings may be attributed to a sicker patient profile and further studies with a larger sample population are warranted to standardize them. The study may provide further guidance for future studies on outcome predictors in COVID-19 patients. The insight gained may guide future vaccination strategies in high-risk groups and hospital development policies with a vision to better fight further waves of this pandemic.
